# Characterizing pharmacogenetic programs using the consolidated framework for implementation research: A structured scoping review

**DOI:** 10.3389/fmed.2022.945352

**Published:** 2022-08-18

**Authors:** John H. McDermott, Stuart Wright, Videha Sharma, William G. Newman, Katherine Payne, Paul Wilson

**Affiliations:** ^1^Manchester Centre for Genomic Medicine, St Mary’s Hospital, Manchester University Hospitals NHS Foundation Trust, Manchester, United Kingdom; ^2^Division of Evolution, Infection and Genomics, School of Biological Sciences, The University of Manchester, Manchester, United Kingdom; ^3^Division of Population Health, Manchester Centre for Health Economics, Health Services Research and Primary Care, School of Health Sciences, The University of Manchester, Manchester, United Kingdom; ^4^Division of Informatics, Centre for Health Informatics, Imaging and Data Science, School of Health Sciences, The University of Manchester, Manchester, United Kingdom; ^5^Division of Population Health, Centre for Primary Care and Health Services Research, Health Services Research and Primary Care, School of Health Sciences, The University of Manchester, Manchester, United Kingdom

**Keywords:** pharmacogenetics, precision medicine, implementation science, medical informatics, pharmacogenomics

## Abstract

Several healthcare organizations have developed pre-emptive pharmacogenetic testing programs, where testing is undertaken prior to the prescription of a medicine. This review characterizes the barriers and facilitators which influenced the development of these programs. A bidirectional citation searching strategy identified relevant publications before a standardized data extraction approach was applied. Publications were grouped by program and data synthesis was undertaken using the Consolidated Framework for Implementation Research (CFIR). 104 publications were identified from 40 programs and 4 multi-center initiatives. 26 (66%) of the programs were based in the United States and 95% in high-income countries. The programs were heterogeneous in their design and scale. The Characteristics of the Intervention, Inner Setting, and Process domains were referenced by 92.5, 80, and 77.5% of programs, respectively. A positive institutional culture, leadership engagement, engaging stakeholders, and the use of clinical champions were frequently described as facilitators to implementation. Clinician self-efficacy, lack of stakeholder knowledge, and the cost of the intervention were commonly cited barriers. Despite variation between the programs, there were several similarities in approach which could be categorized *via* the CFIR. These form a resource for organizations planning the development of pharmacogenetic programs, highlighting key facilitators which can be leveraged to promote successful implementation.

## Introduction

Medicines are the most common therapeutic intervention in healthcare, yet their effectiveness and safety show considerable inter-personal variation (1, 2). Such variation is regularly attributed to the chosen dosing strategy, the accuracy of the initial diagnosis or individual factors, such as co-morbidities, polypharmacy or adherence. In addition, there is an increasing awareness that response to medicine is affected by an individual’s genetic variation, a concept known as pharmacogenetics. This has a significant personal, clinical, and financial impact, leading to poorer individual and population-level outcomes and waste of scarce healthcare resources (3, 4). Using the results of a pharmacogenetic test, embedded in a model of service delivery to inform prescribing decisions (hereafter “pharmacogenetics”) could lead to more accurate medicine selection and dosing, improving patient outcomes and better use of healthcare budgets (5).

Evidence-based guidelines to support pharmacogenetics are available for many commonly prescribed medicines (6–9). Despite a good understanding of these medicine-gene relationships, implementation of pharmacogenetics into clinical practice in many countries is limited to a small number of drug-gene pairs. This is typically carried out where variants in single genes are genotyped at the point of prescription (hereafter “reactive testing”) (10). Given the high population frequency of genetic variation which influences the effectiveness and safety of medicines, an alternative to reactive-testing is pre-emptive panel testing (11). This involves testing individuals for many common pharmacogenetic variants at a set time, irrespective of the medicine they are prescribed (hereafter “pre-emptive testing”). This information can then be stored in medical records and used to inform life-long prescription.

**FIGURE 1:**
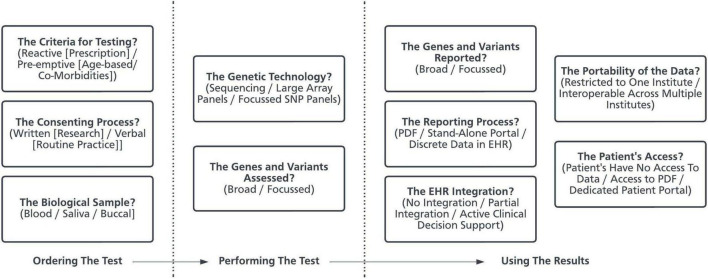
The decisions made when developing a pharmacogenetic program. Using pharmacogenetics in clinical practice is a complex healthcare intervention and is made up of many components (16, 80). This schematic presents some of the key design decisions made by organizations when developing a pharmacogenetic service. PDF, Portable Document Format. EHR, Electronic Health Record. SNP, Single Nucleotide Polymorphisms.

In several countries there is increasing interest in integrating pre-emptive pharmacogenetic testing into routine practice (12, 13). This represents a complex healthcare intervention comprising many interacting components and, as such, several factors influence the likelihood of successful implementation (14). Implementation is defined as the process of integrating evidence-based interventions within a setting (15). Pharmacogenetics represents one such evidence-based intervention, and the “implementation of pharmacogenetics” can be defined as the process by which gene-drug prescribing guidelines (the evidence base) are realized to guide prescribing for patients. Pharmacogenetic programs represent the organizational structures which aim to implement pharmacogenetics in practice. At present, there is no consensus around how pre-emptive pharmacogenetic programs should be designed. It is highly probable that programs designed to deliver pre-emptive pharmacogenetics will differ in their optimal design depending on the clinical and institutional context. Despite this, there are certain design decisions and methodological challenges which are common across programs (Figure 1) (16). The choices made when setting up individual programs, in response to individual organizational requirements, will result in varied approaches to implementation.

Structured conceptual frameworks provide an approach by which heterogeneous programs can be examined to systematically identify factors which influence implementation (17). Grounded in Diffusion of Innovations theory and widely empirically tested, the Consolidated Framework for Implementation Research (CFIR) is one such framework which can be applied systematically to identify factors that may emerge in various, multi-level contexts to influence implementation (17–19). Characterizing the barriers and facilitators which influenced the development of existing programs to implement pharmacogenetics can support and expedite the formation and development of new and existing initiatives. Common themes which appear to predict success can be included, whilst recurrent barriers can be addressed and overcome. This work identifies and synthesizes the literature describing the application of pre-emptive pharmacogenetics in clinical practice, characterizing the barriers and facilitators to implementation using the CFIR.

## Methods

A structured literature review was undertaken to summarize the key characteristics of a pre-emptive pharmacogenetic testing program. A bidirectional citation search was used to systematically identify publications of interest followed by evidence-synthesis using the CFIR (20, 21).

### Research aim

The study aimed to characterize the barriers and facilitators to the implementation of pre-emptive pharmacogenetics.

### Identification of literature pool

#### Inclusion criteria

Original research published in peer-reviewed journals describing the implementation of a multi-gene (>1 gene) pharmacogenetic panel in clinical practice. For the purposes of this analysis, implementation was considered to have taken place where the results of a pharmacogenetic test were used to influence prescribing behavior. This included both clinical activity and research projects, where testing and prescribing was performed under research ethics.

#### Exclusion criteria

Systematic reviews or opinion-pieces were excluded. Original studies referenced in identified systematic reviews were identified using hand-searching of the reference lists and included. Conference abstracts and non-English language studies were also excluded.

#### Search strategy

A Bidirectional Citation Search was used to identify the literature of interest (20). This is a literature searching strategy which provides a systematic approach to maximize the identification of relevant articles in interdisciplinary topics. The starting pool (“initial pearls”) were identified via a pragmatic Boolean search of Medical Subject Heading (MeSH) Terms within Web of Science; MEDLINE (*via* OVID); Embase (*via* OVID); and PubMed Central on 1 February 2022. The following search [(((Pharmacogenetics) OR (Pharmacogenomics)) AND (Implementation)) AND (pre-emptive)] was used. No date limits were used. For each article, preliminary relevance was determined by review of the title and abstract. Articles were chosen as the initial pearls based on their breadth and quality, determined in part by pre-existing awareness of the literature and ensuring balance between articles describing research in primary and secondary care. Relevant articles known to the study team which were not identified *via* the Boolean search were added as further initial pearls. The collation of papers was conducted first by one author (JM) but, to assess the validity of article selection, 25% of the candidate papers was reviewed by a second author (SW). Inter-rater reliability was assessed *via* Cohen’s Kappa (κ) statistic.

#### Data extraction

Titles and abstracts of studies identified from the initial Boolean search and at each stage of the bidirectional citation search were screened for eligibility. Those studies deemed suitable for inclusion were assessed using a standardized *pro forma* (Supplementary Appendix), which included a section dedicated to the CFIR. The CFIR comprises a comprehensive taxonomy across five domains that are likely to influence the implementation of any innovation. These are:

I Intervention characteristics—the features of the innovation that may influence implementation.
II Inner setting—the ways by which organizational leadership, culture and delivery can influence implementation.
III Outer setting—the wider contextual or policy factors that might influence implementation and sustainability.
IV The characteristics, beliefs and attitudes of the individuals involved in implementation.
V How the process of implementation is actually enacted.

These domains are made up of different constructs and sub-constructs, each of which relate to a facet of implementation. Detailed definitions of the CFIR domains and constructs are provided in Supplementary Table 1.

**FIGURE 2:**
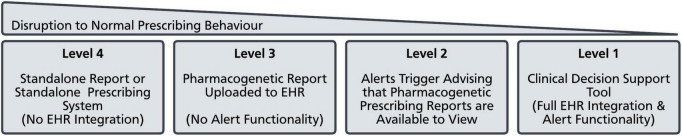
Approaches to deliver pharmacogenetic data to prescribers. Approaches to deliver pharmacogenetic data to prescribers were grouped into 4 categories which varied in how much they disrupted normal prescribing behavior. EHR, Electronic Health Record.

Data extracted included geographic location, clinical setting, participants, ethical framework for undertaking pharmacogenetic testing, chosen testing strategy, integration with the Electronic Health Record (EHR), and mapping to the CFIR. To systematically categorize approaches to return pharmacogenetic test results to prescribers, a pragmatic classification system was developed which included 4 levels, ranging from the most (Level 4) to least (Level 1) disruption to routine prescribing behavior (Figure 2). Each manuscript was reviewed to assess whether a given CFIR domain was referred to. If present, the specific constructs were mapped, and a narrative summary of each domain undertaken. Whether the construct was referenced as a barrier or a facilitator to implementation was also recorded.

#### Data synthesis

Where several manuscripts described different facets of implementation at the same program, or described the development of a program over time, these were grouped together for analysis to provide an overview of the barriers and facilitators of pharmacogenetics at that specific program. Details of the geography, clinical setting, and participants of each program were summarized. A narrative summary was conducted to review the organizational and ethical frameworks used to undertake testing, specifically focusing on the criteria for testing and whether testing was undertaken on a purely clinical basis (requiring standard clinical consent only) or on a research basis (requiring research consent). Further narrative summaries were also performed to assess the chosen testing strategies, and the approaches to return test results to clinicians. A narrative summary was also constructed for each of the CFIR domains.

## Results

### The literature pool

**FIGURE 3:**
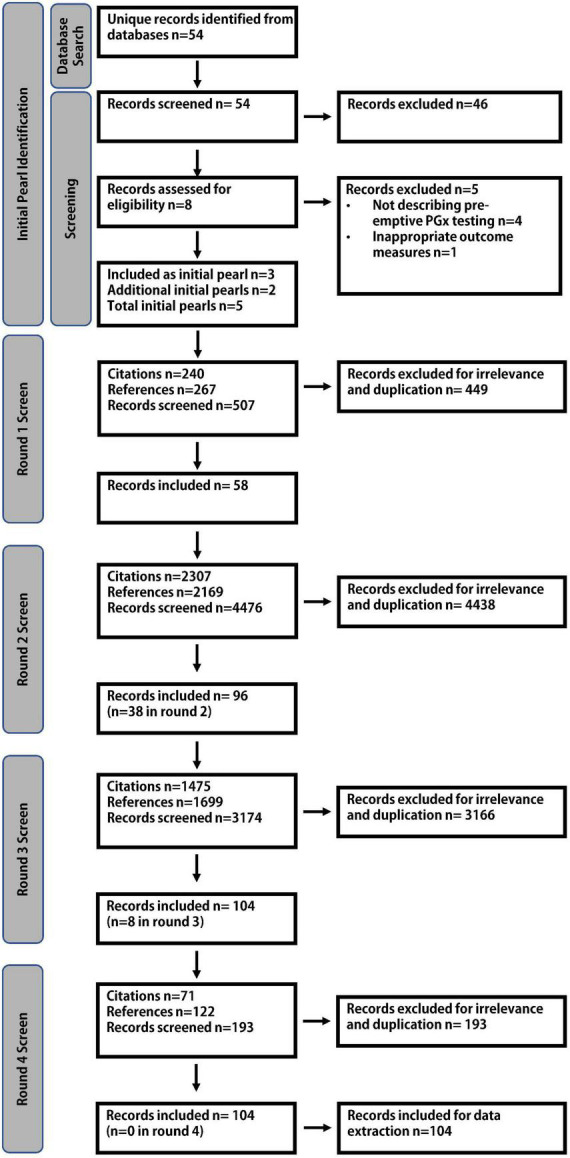
Literature search strategy. An initial Boolean literature search resulted in 5 initial pearls. Four rounds of screening were required to complete the bidirectional citation search, identifying 104 relevant publications.

Fifty-four unique articles were retrieved *via* the Boolean search and underwent independent title and abstract review by two authors (JHM and SW). Eight underwent full text review and 3 were chosen as initial pearls (Figure 3). Based on a pre-existing awareness of the literature, 2 further articles were added to the literature pool, resulting in 5 initial pearls entering the bidirectional citation search, agreed upon by all authors (22–26). Four screening rounds were required to complete the bidirectional citation search which involved the review of 8,355 abstracts. 104 relevant publications were identified for analysis (Figure 3). The inter-rater reliability showed 96.7% agreement with a Cohen’s kappa statistic of 0.71, representing substantial agreement.

### Characteristics of the pharmacogenetic literature

The 104 publications mapped to 40 distinct programs and 4 multi-center initiatives, namely the US based Implementing GeNomics In pracTticE (IGNITE) and Electronic Medical Records and Genomics (eMERGE) programs, the international Pharmacogenomics Global Research Network (PGRN) initiative and the European U-PGX project (Supplementary Table 2). 26 (66%) of the programs were based in the United States, whilst Spain (3) and the Netherlands (2) had the greatest number of independent programs in Europe (Supplementary Figure 1 and Supplementary Table 2). Only 2 (5%) of programs were based in middle-income countries. The remaining 38 (95%) were located in high-income countries. Programs varied considerably in their scale and complexity. Some programs were referenced by multiple manuscripts, whereas others were described by a single manuscript only (range 1–10).

#### The eligibility for pharmacogenetic testing

Several organizations implemented pharmacogenetics within the context of a research trial, requiring formal written consent approved under research ethics. The structure and governance of these trials varied and, in part, was prescribed by the prevailing regulatory landscape for conducting clinical research (27, 28). Some of these studies were focused on specific populations or disease states as part of the research design. The Toronto based IMPACT study undertook testing in individuals treated with psychotropic medication, whilst the University of Chicago’s ImPreSS Trial aims to explore the usefulness of pre-emptive pharmacogenetic testing in the peri-operative setting (29, 30). Some initiatives recruited patients in certain age groups, the PHARM-GENOME-PACE study recruiting participants over the age of 55 years, and the University of Utah exploring benefits in those aged over 65 years of age (31, 32).

Several programs leveraged existing biobanks to implement pharmacogenetics. Mount Sinai Hospital’s CLIPMERGE PGx initiative enrolled patients to BioMe, an existing electronic health record (EHR) linked biobank (33). Similarly, The Mayo Clinic recruited participants from the Mayo Clinic Biobank for the Right Drug, Right Dose, Right Time (RIGHT) Trial (34). The University of Colorado also adopted a comparable approach, using the Colorado Center for Personalized Medicine Biobank to support its pharmacogenetic service (35).

Although some institutes implemented pharmacogenetics through research protocols with strict eligibility criteria, others developed a strategy where widely available pharmacogenetic programs were iteratively assessed *via* research methodologies. This is best exemplified at St Jude Children’s Hospitals, where pharmacogenetic testing is available for patients without restrictive eligibility criteria, though this still operates under research ethics (36). The University of Florida have utilized a similar model, although with some key differences. The institute has developed a pre-emptive pharmacogenetic gene panel which does not require research ethics, but the institute also engages in more targeted pharmacogenetic testing for specific indications such as selective serotonin reuptake inhibitors (SSRIs), opioid and Proton Pump Inhibitor (PPI) prescribing (37, 38). This more targeted testing has been undertaken under the auspices of research ethics, as part of pragmatic randomized trials.

There were several examples of pharmacogenetic programs which were undertaken on a clinical basis with no research consent required, rather than requiring distinct ethical approval. Most notably, the PREDICT project at Vanderbilt University Medical Center (VUMC), which has tested well over 10,000 patients, was designed as a quality improvement project, with the aim of better implementing FDA guidance for prescribing (39). During the initial design phase the PREDICT steering group, recognizing the seminal nature of the program, asked the local ethics committee to review the proposal to assess for any ethical concerns. As such, there is no formal informed consent process, beyond what would be expected as part of standard clinical practice, for pharmacogenetic testing at VUMC. Importantly, this approach has not impacted the PREDICT’s team’s ability to make major contributions to the pharmacogenetic literature (39, 40).

#### The genetic technology

An important component of any pharmacogenetic program is the choice of genetic technology—specifically the laboratory test which is able to identify whether the patient carries clinically relevant variants. There were differences between programs both in relation to the genes and variants tested (hereafter “targets”), and also with regard to the genetic technology used (Supplementary Table 2). However, the majority of programs adopted array-based platforms (26, 36, 41–46). These ranged from extremely broad systems, with the capacity to flexibly test for many thousands of variants across hundreds of genes, to more targeted gene panels (35). Where a service was designed around a specific indication, such as mental health or hypertension, panels were occasionally restricted to include genes and variants associated with medicines related to those diseases only (47–49). Array platforms from several commercial vendors were used by pharmacogenetic programs, and most systems allowed for an element of customization. These results demonstrate a lack of standardization in both panel design and platform.

Despite variability in the choice of test provider and target selection, almost all centers made use of genotyping rather than sequencing approaches, though there were two exceptions. The Hospital for Sick Children in Toronto, as part of a clinical trial, compared pre-emptive against reactive-testing (50). Pre-emptive pharmacogenetic testing involved the reanalysis of whole genome sequencing (WGS) data undertaken as part of separate investigations for congenital cardiac malformations. The Mayo Clinic developed PGRNseq for the RIGHT Study which is a Targeted Capture Sequencing Panel for over 250 genes (34, 41, 51).

#### Electronic health record integration

There were several strategies for how pharmacogenetic data was returned to be used at the point of prescribing to enable informed treatment choices. These were categorized into four main approaches which vary in their complexity and impact on typical prescribing behavior (Figure 2). The most disruptive approach (Level 4) was to produce a standalone report with no integration into the patient’s EHR. These reports were typically sent to the requesting clinician as PDF documents *via* e-mail. A variant of this approach was to develop a standalone pharmacogenetic interface which clinicians could interact with externally to their main EHR. Academics at the University of British Colombia developed a medication decision support system (MDDS) which *via* a series of logic trees would consider several patient variables, including pharmacogenetic variation, and recommend optimal therapeutic approaches for specific conditions (35). Critically, this was a distinct piece of software and not built into existing electronic prescribing tools. Most centers developed at least some level of integration with their EHR. The least complex approach was to upload a copy of the pharmacogenetics report to the EHR (Level 3), so it was available to view by the prescriber. The reports were predominantly uploaded as Portable Document Format (PDF) documents, though some centers were able to upload the results as discrete data.

Several programs have achieved more sophisticated levels of integration within their EHRs, and a number of manuscripts are dedicated to describing the technical methodologies these centers have utilized (42, 52–55). The general approach was to make use of EHR clinical decision support (CDS) functionality, specifically alert based decision support tools. In the EPIC^®^ EHR, these are known as best practice advisories (BPAs). These would trigger under certain conditions and could be used to facilitate pharmacogenetic guided prescribing. The Cincinnati Children’s Hospital was initially unable to store pharmacogenetic data as a discrete variable within the EHR, meaning that drug-variant BPAs could not be developed (48). Instead, they developed an advisory alert indicating pharmacogenetic results were available within the EHR when certain medicines were prescribed (Level 2). This would then require the prescriber to search for the stored results if they wished to make use of them. Where organizations were able to store pharmacogenetic results as a discrete variable within their EHR, they were able to make use of active BPAs (Level 1) (36, 40, 42, 56–61). These would trigger when a drug was prescribed where there was a clinically actionable pharmacogenetic prescribing recommendation available.

### Characterizing pharmacogenetic programs via the consolidated framework for implementation research

**FIGURE 4:**
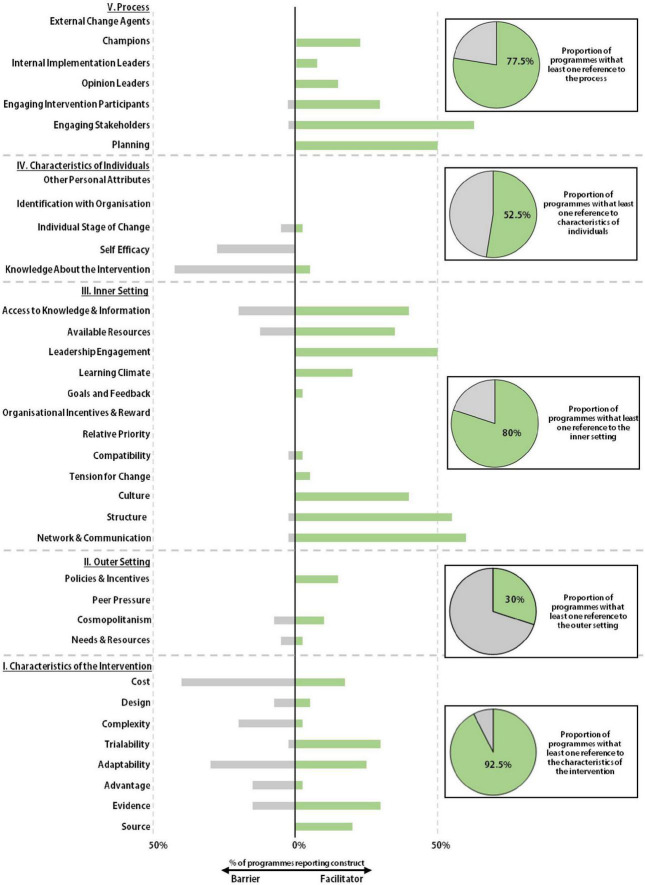
Facilitators and barriers to implementation. The Consolidated Framework for Implementation Research (CFIR) was used as a framework to identify the barriers and facilitators to implementation at each pharmacogenetic program. Whether a program referenced any construct within a given domain was recorded and displayed as a circle graph. Referenced CFIR constructs were recorded as being described as either a barrier or facilitator to implementation and displayed onto the stacked bar chart.

Not all programs were described in the same level of detail. The implementation efforts at some organizations were clearly described, with multiple manuscripts detailing their development over time (Supplementary Figure 2). Others meanwhile were described by single manuscripts with less detail. Comparing reporting within and between each CFIR domain meant that data from all programs delivering pharmacogenetics could be extracted and synthesized, highlighting overall barriers and facilitators in the identified literature. Certain domains and constructs were referenced significantly more frequently than others (Table 1 and Figure 4).

**TABLE 1:** The most highly referenced CFIR constructs.

Domain	Construct	Frequency referenced (%) and rank (*n* of 37)	Key considerations when designing a future pharmacogenetic program
I. Intervention	Cost	57.5% (5)	Cost is a barrier—economic analysis may be required to support implementation in some health systems
	Adaptability	55.0% (6)	The intervention should be designed to disrupt existing prescribing practice as little as possible
	Evidence	45.0% (10)	Evidence for gene-drug pairs should be clear and well communicated to clinical stakeholders
	Trialability	32.5% (12)	The intervention should be iterated over time to identify issues and build stakeholder confidence
III. Inner setting	Structure	62.5% (2)	Pilot centers for new programs should be chosen based on their existing academic expertise and experience of organizational innovation
	Access to knowledge and information	60.0% (3)	Clinical stakeholders should be provided digestible information about the program and how to incorporate it into their work
	Networks and communication	57.5% (4)	Prior to implementation, clear organizational structures and lines of communication should be established between key stakeholders
	Available resources	47.5% (8)	Programs require resources for implementation and on-going operations including money, training, education, physical space, and time
	Culture	40.0% (11)	Pilot centers should have cultures which promote and embrace change
IV. Characteristics of individuals	Knowledge and beliefs about the intervention	47.5% (9)	Efforts should be made to educate clinical stakeholders on the relevance of pharmacogenetics to their own practice
	Self-efficacy	27.5% (14)	Clinical stakeholders should be educated and empowered to make use of pharmacogenetic guided prescribing
V. Process	Engaging stakeholders	65.0% (1)	Clinical stakeholders should be engaged and educated early, making use of varied resource including asynchronous and “just in time” learning
	Planning	50.0% (7)	Multi-disciplinary oversight boards should be established to organize and oversee the development of a program
	Engaging intervention participants	32.5% (13)	Public stakeholders should be meaningfully involved in the development of the program and consideration should be given to developing patient facing pharmacogenetic tools, allowing access to data

The 15 most highly referenced Consolidated Framework for Implementation Research (CFIR) constructs are presented and relevant considerations for the development of a new pharmacogenetic service are discussed.

#### Domain I: Characteristics of the intervention

The Characteristics of the Intervention domain is comprised of eight constructs (Figure 4 and Supplementary Figure 3). Each intervention construct was cited by at least one of the identified programs and 92.5% of all programs made at least one reference to a construct within the domain. Adaptability was one of the most frequently mapped CFIR domains, discussed by 55% of all programs, 30% describing the construct as a barrier and 25% referencing it as a facilitator. In the context of a pharmacogenetic service, adaptability relates to how pharmacogenetic guided prescribing can be implemented without disrupting normal clinical practice. Authors from several institutes noted that tailoring their service to existing clinical practice resulted in higher levels of satisfaction amongst stakeholders.

The Eskenazi Health System, as part of the INGENIOUS trial, developed a pharmacogenetic consult service, analogous to existing pathology consultations in their institution (62). Where required, physicians could request support with pharmacogenetic guided prescribing, making use of established clinical pathways. Several programs were able to integrate pharmacogenetic data directly into their local EHRs, thus facilitating implementation. Centers which were unable to integrate results into their local EHRs *via* clinical decision support tools were more likely to discuss adaptability as a challenge or barrier to integration (63). One study which did not have BPA functionality noted that “developing CDS tools is critical for the clinical utility to parallel the longevity of PGx results,” whilst another concluded that “current electronic and paper laboratory test information stores relegate historical test findings to an ‘out of mind’ position for most clinicians” (47).

Cost was the most frequently discussed construct within the intervention domain and was overwhelmingly cited as a barrier to implementation, with 46% of programs discussing cost as a barrier. This barrier is related to the absolute dollar price of the test but also, predominantly in the US market, is related to the complexities of navigating the health insurance system where there is considerable variation in coverage and a lack of transparency around which tests can be ordered (64). 32% of programs discussed the strong evidence base which exists to support the implementation of pharmacogenetics as a facilitator, whilst 22% of programs noted stakeholders responded positively to the system being developed by their own institution. 31 (77.5%) of the programs made reference to CPIC in at least one publication, demonstrating the importance of these guidelines. However, it was not possible to directly assess how these guidelines impacted the design of the pharmacogenetic programs or the uptake from clinical stakeholders.

#### Domain II: Outer setting

The Outer Setting relates to the wider contextual or policy factors that might influence implementation and sustainability. This was one of the least referenced CFIR domains in the literature pool (Figure 4). Only 30% of programs referred to this domain, in contrast to the Inner Setting which nearly 80% of programs referred to at least once. The most frequent reference to the Outer Setting was in relation to the Policies and Incentives domain which was referenced by 15% of programs. This was typically in relation to US national collaboratives such as the IGNITE network and e-MERGE. Some organizations described their involvement in these groups and noted how they stimulated implementation activity and supported the sharing of best practice (53, 65). The concept of cosmopolitanism, the degree to which a pharmacogenetic program is networked with external organizations, was referred to by 18% of programs, meaning the scalability of pharmacogenetic data across organizations was not a commonly reported design attribute.

#### Domain III: Inner setting

The Inner Setting Domain within the CFIR considers whether an institute has the structure, institutional climate and the networks and communication to support implementation. It also considers an institution’s readiness for implementation, which corresponds to leadership engagement, the availability of resources to support implementation, and stakeholder access to relevant educational material. Many centers established educational programs and developed dedicated programs or departments to support implementation (26, 66, 67). A positive learning climate was described by 35% of programs and ready access to information was positively referenced by 40% of institutes. Where stakeholders were not provided with sufficient access to knowledge and information (20% of programs), or resources were not readily available (13% of programs), this was viewed as a barrier to implementation as clinicians struggled to interpret and therefore, implement the pharmacogenetic data.

Support from the host institution, whether fiscal, organizational, or simply prioritization of the initiative, was seen as a facilitator to the development of a successful implementation program. The Mayo Clinic, Vanderbilt University Hospitals and St. Jude Children’s Hospitals are three leading centers for pharmacogenetics. These programs are by no means identical, but there are notable similarities in their development. All three organizations formed internal program committees, with stakeholders from different specialities to oversee the growth and implementation of their service (36, 40, 68). Early leadership engagement was seen as one of the most important facilitators to the development and ongoing operation of a pharmacogenetic service, referenced by 50% of all programs.

Many pharmacogenetic services have been developed at large academic hospitals. These organizations are typically fertile environments for the implementation of new services, and this type of innovation is embedded into their institutional culture with 40% of programs referencing a supportive institutional environment as a facilitator. 60% of programs referenced the value of effective communication networks, whilst 55% of programs made note of the intrinsic structural characteristics of their institute (maturity, academic expertise, size etc.) as being important to successful implementation.

#### Domain IV: Characteristics of the individual

A total of 52.5% of all programs referred to a construct within the Characteristics of The Individual domain at least once (Figure 4). When referenced, constructs within this domain were mostly described as barriers to implementation. Knowledge and Beliefs about the Intervention, was a frequently considered construct within the pharmacogenetic literature, referred to by 48% of all programs. The majority of these programs highlighted this construct as a potential barrier to implementation. In an institutional profile of the Colorado Center for Personalized Medicine, the authors state that clinical stakeholders questioned the rationale for their testing approach and noted that “conversations about the pharmacogenomic initiative were side-tracked by concerns about genetic testing, which were largely a result of stakeholders’ experiences with genetic testing (for rare disease)” (35). The authors of several manuscripts across multiple organizations noted that real world evidence to support the widespread implementation of pharmacogenetics is somewhat variable, which could also impact beliefs about the utility of the intervention. A number of more recent manuscripts have begun to address this lack of evidence (51).

To improve clinical stakeholders’ knowledge and beliefs about the use of pharmacogenetics in clinical practice, several centers established educational programs. In 2017, the e-MERGE network published an overview of the educational programs operated by its 10 member organizations (69). All of the sites recognized the need to educate clinicians about pharmacogenetics either within the context of specific studies or as part of deploying CDS tools within the EHR. The educational strategies deployed by different programs varied in their scale, content and methodological approach (66, 67). Several sites made use of online resources which clinicians could access in their own time to support asynchronous learning. Some sites, such as the Mayo and Sanford Health, also offered more structured certificates to educate the workforce.

One major aim of these educational programs was to improve a clinician’s self-efficacy which, in the context of pharmacogenetics, refers to an individual’s confidence to perform pharmacogenetic guided prescribing *via* their institutional program. Self-efficacy, specifically clinician awareness or understanding of how to apply the pharmacogenetic data, was highlighted as barrier by 28% of programs (26, 70–73). Whether self-efficacy was improved by the educational initiatives can be inferred, to an extent, by the longevity and success of many initiatives, though this was not formally measured. Indeed, there are few studies which directly assess the impact of these programs on individual behavior. Rather, they focus on the global change in practice within an institution. An Individual’s Identification with the Organization and the Individual’s Stage of Change are two of the least mapped CFIR constructs within the pharmacogenetic literature, with no references identified during this study.

#### Domain V: Process

The Process domain can be summarized as how change has been enacted at a given institution. 77.5% of programs referenced at least one construct from within this domain. At many centers, steering committees were established to oversee development of pharmacogenetics programs. These groups were tasked with designing and implementing a program within an organization, meaning their effective functioning could be a powerful facilitator to successful implementation. This planning period appears to have been important in the development of several programs and was referred to by 50% of all institutes. When the Cincinnati Children’s Hospital Medical Center launched their pharmacogenetics program, they established a multi-disciplinary committee with representation from a broad range of specialities (48). The group would meet regularly to discuss all aspects of the program’s development and were able to cascade information back to their own clinical teams, promoting stakeholder engagement, the most consistently mapped construct within the pharmacogenetic literature, referenced as a facilitator by 63% of programs.

Engaging stakeholders, both clinical and non-clinical, is an important part of any program and impacts how an organization promotes adoption of the intervention. This can take the form of clinical representation on steering groups, as discussed above, but also includes the establishment of educational programs or the appointment of clinical champions. These individuals, typically chosen from within the organization, contribute to the implementation of an intervention through early adoption, promotion, and marketing. 23% of all programs referenced their use of clinical champions as a facilitator to implementation. A retrospective review of the Mission Health Personalized Medicine Program found that the use of physician champions, and ensuring they were supportive of the program in each speciality, was one of the most significant factors in the success of the program (74).

## Discussion

This study has identified several key barriers and facilitators important to the development and implementation of pharmacogenetics. Using the CFIR, these constructs have been categorized into domains and constructs which highlight similarities between programs. The findings from this study may be beneficial to individual organizations or health care systems when developing programs in the future.

This review has identified a number of constructs which existing programs consistently cite, or fail to cite, which could highlight potential areas for focus within other healthcare systems (Table 1). Many programs had strong institutional support, early leadership engagement and learning cultures suited toward implementation. This will be important within any new program. However, whereas individual centers can manage their institutional eco-systems closely and foster support for implementation, this is more challenging across larger and more complex healthcare systems, such as in the United Kingdom. In nationally co-ordinated healthcare systems, there is no mechanism for individual hospitals to develop their own discrete pharmacogenetic program, as is the case in the US. This provides an argument to suggest that, within such healthcare systems, there should be support for early-adopter centers, or regional hubs, which can develop exemplar services followed by iterative expansion across the wider system. This would facilitate the trialability of pharmacogenetics, a frequently identified construct throughout the pharmacogenetic literature.

The Outer Setting was one of the least referenced domains within the literature. Identified manuscripts overwhelmingly focused on the development of a pharmacogenetic service at a single institution, rather than across a state-wide or national healthcare system. As such, the portability of pharmacogenetic data between different settings, or different EHRs, was not an essential consideration. This has obvious disadvantages where a patient’s care might be managed across various organizations, resulting in a need to share data across institutional boundaries. This is especially the case in healthcare systems where patients routinely transition between hospitals and primary care providers for their management. Even in the US, where many patients might receive a large proportion of their care from a single provider, there remains notable fragmentation for some users, disproportionally affecting vulnerable populations (75, 76). As such, in many healthcare settings the concept of Cosmopolitanism should be an essential design consideration. Designing an IT solution which can support pharmacogenetic guided prescribing for clinicians in an effective and equitable way across a healthcare system is a tractable problem, but one which will require considerable collaboration and effort. The data presented here suggests that launching programs to deliver pharmacogenetics without such a solution would be a significant barrier to uptake and reduce the potential long-term benefits, failing patients and clinicians.

It should be noted that domains and constructs within the CFIR do not exist in isolation of each other. Theoretical frameworks such as the CFIR are useful tools to catalog barriers and facilitators within implementation programs, but careful interpretation of these mapping exercises is required to make informed connections between the mapped domains. For example, the design of a pharmacogenetic prescribing system and the strength of evidence supporting the intervention will both impact on stakeholder engagement. Although these are separate constructs within the Characteristics of the Implementation domain, their impact in combination is arguably greater than their individual relevance. For example, the evidence collated within this review suggests that a well evidenced gene-drug interaction has little chance of successful implementation without a properly designed EHR system to deliver the pharmacogenetic data to prescribers in a usable format at the point of use. Equally, a well-designed EHR has limited usefulness if there is only poor evidence for a specific gene-drug pair. Furthermore many constructs will be positively correlated. For example, a positive institutional culture, which promotes the adoption of new technologies, is very likely to be correlated with an effective organizational structure with strong networks of communication as was the case for several leading pharmacogenetic programs.

The IGNITE network has previously surveyed their member organizations and used the CFIR as a tool to categorize and assess their responses (77–79). When IGNITE project teams were asked to rate the CFIR constructs in order of perceived value for genomic medicine, the findings were consistent with those identified in this study (77). No high-priority constructs were identified in the Outer Setting, whilst engaging stakeholders, a positive institutional culture and individual knowledge and beliefs were recognized as important constructs. A similar exercise, undertaken with IGNITE members who had implemented pharmacogenetic guided prescribing for antidepressants, asked stakeholders to choose the CFIR constructs considered “most important” in each domain. There is a good correlation between these “most important” constructs, and the recurrent constructs identified in this work (Table 1). Where inconsistencies exist, they are most likely explained by the different methodologies used to identify key constructs. The IGNITE studies made use of survey-based methods, whereas this work utilized a retrospective analysis of the published literature. Each approach has its advantages and limitations, but the consistency in constructs considered importance in these manuscripts suggests the findings can be considered reliable.

This study has limitations which are important to consider when interpreting the extracted data. Firstly, the identified manuscripts are subject to survivor bias. Some organizations may have chosen not to publish manuscripts detailing the development of their programs and the ones that have may skew toward larger academic health centers which may not be entirely representative of the pharmacogenetic implementation initiatives. Furthermore, there may be a reporting bias in how institutes describe their programs. Programs may not have disclosed some of the barriers they faced or may have made the decision to focus on certain aspects of implementation over others. For example, although we have interpreted the lack of reference to the Outer Setting as a sign that this was less frequently considered by programs, it is possible that this it is routinely considered though less frequently reported in the literature. As such, other research methodologies such as semi-structured interviews should be used to supplement and corroborate these data in the future. Finally, the majority of identified programs were US based and are unlikely to be entirely comparable to how pharmacogenetic services might be developed in other healthcare systems.

## Conclusion

This study identified a large number of programs to deliver pharmacogenetics with varying designs across a wide geography and timespan. Despite heterogeneity, there were a number of similarities identified which could be categorized using a structured implementation framework. These form a useful resource for organizations approaching the development of pharmacogenetic services. Early engagement of stakeholders, developing a fertile implementation climate, the development of adaptable IT solutions and an iterative approach to implementation are all fundamental constructs which should be considered when implementing pharmacogenetics.

## Data availability statement

The original contributions presented in this study are included in the article/Supplementary material, further inquiries can be directed to the corresponding author/s.

## Author contributions

JM and PW developed the concept for the manuscript. JM and SW identified and reviewed the literature. JM undertook the analysis with support of SW, WN, and PW. JM drafted the manuscript with critical review from all authors. All authors read and approved the final version of this manuscript and contributed to the development of the methodology.
