# Supplementary material for: Characterizing pharmacogenetic programs using the consolidated framework for implementation research: A structured scoping review
Source: Front Med (Lausanne). 2022 Aug 18;9:945352. doi: 10.3389/fmed.2022.945352 (PMC9433561; doi:10.3389/fmed.2022.945352)
Supplement: Supplementary file 2 [file Data_Sheet_2.docx]

**Supplementary Information:** **Characterizing Pharmacogenetic Programmes Using the Consolidated Framework for Implementation Research: A Structured Scoping Review**

**Contents**

1. **Data Extraction Tools**
2. **Table S1: CFIR Domains and Constructs**
3. **Table S2: Pharmacogenetic Testing Programmes and Initiatives**
4. **Supplementary Figures**

**Figure S1. Geographical Distribution of Pharmacogenetic Programmes.**

**Figure S2. Chronology of the Pharmacogenetic Literature.**

**Figure S3. The Consolidated Framework for Implementation Research.**

1. **Data Extraction Tools**
2. **Demographic Details of Each Programme**

1. **Proforma 2: CFIR Constructs Referenced in Each Manuscript**

1. **CFIR Domains and Constructs**

| **Construct** | | **Short Description** |
| --- | --- | --- |
| **I. INTERVENTION CHARACTERISTICS** | | Description (*Contextualised for* *Pharmacogenetics)* |
| A | Intervention Source | Perception of key stakeholders about whether the intervention is externally or internally developed.  *(Was the pharmacogenetic programme developed at the institution where it is being implemented?)* |
| B | Evidence Strength & Quality | Stakeholders’ perceptions of the quality and validity of evidence supporting the belief that the intervention will have desired outcomes.  *(Do stakeholders perceive that there is sufficient evidence for the implementation of pharmacogenetic guided prescribing in their speciality)* |
| C | Relative Advantage | Stakeholders’ perception of the advantage of implementing the intervention versus an alternative solution.  *(Do stakeholders perceive that implementing pharmacogenetics in their speciality would lead to improve patient outcomes and/or cost savings)* |
| D | Adaptability | The degree to which an intervention can be adapted, tailored, refined, or reinvented to meet local needs.  *(How closely does the proposed pharmacogenetic guided prescribing approach resemble or integrate with current practices?)* |
| E | Trialability | The ability to test the intervention on a small scale in the organization, and to be able to reverse course (undo implementation) if warranted.  *(Can the pharmacogenetic programme be piloted in the institution prior to widespread implementation?)* |
| F | Complexity | Perceived difficulty of implementation, reflected by duration, scope, radicalness, disruptiveness, centrality, and intricacy and number of steps required to implement.  *(How challenging is pharmacogenetic guided prescribing to perform within the new programme?)* |
| G | Design Quality & Packaging | Perceived excellence in how the intervention is bundled, presented, and assembled.  *(Is the pharmacogenetic guided prescribing system easy to interact with and does it resemble existing design architecture?)* |
| H | Cost | Costs of the intervention and costs associated with implementing the intervention including investment, supply, and opportunity costs.  *(How much does the pharmacogenetics programme cost and/or is their health economic evidence supporting its implementation)* |
| **II. OUTER SETTING** | |  |
| A | Patient Needs & Resources | The extent to which patient needs, as well as barriers and facilitators to meet those needs, are accurately known and prioritized by the organization.  *(Has the pharmacogenetic programme been designed taking into account patient needs and resources?)* |
| B | Cosmopolitanism | The degree to which an organization is networked with other external organizations.  *(Is the pharmacogenetic data available in other healthcare settings, beyond the primary healthcare provider?)* |
| C | Peer Pressure | Mimetic or competitive pressure to implement an intervention; typically because most or other key peer or competing organizations have already implemented or are in a bid for a competitive edge.  *(Are there other local or comparator institutions with a pharmacogenetic service which have driven implementation?)* |
| D | External Policy & Incentives | A broad construct that includes external strategies to spread interventions, including policy and regulations (governmental or other central entity), external mandates, recommendations and guidelines, pay-for-performance, collaboratives, and public or benchmark reporting.  *(1. Are there regional or national guidelines which support the implementation of pharmacogenetics at the institution? 2. Is the institution part of regional or national organisations which can support the implementation of pharmacogenetics (Such as IGNITE or e-MERGE))* |
| **III. INNER SETTING** | |  |
| A | Structural Characteristics | The social architecture, age, maturity, and size of an organization.  *(The social architecture, age, maturity, and size of the organization considering the implementation of Pharmacogenetics)* |
| B | Networks & Communications | The nature and quality of webs of social networks and the nature and quality of formal and informal communications within an organization.  *(How straightforward is it to communicate and share best practice, in relation to pharmacogenetics, at the institution?)* |
| C | Culture | Norms, values, and basic assumptions of a given organization.  *(Is the culture of the organisation supportive of large implementation initiatives, such as would be required for a pharmacogenetic programme)* |
| D | Implementation Climate | The absorptive capacity for change, shared receptivity of involved individuals to an intervention, and the extent to which use of that intervention will be rewarded, supported, and expected within their organization.  *(Does the organisation promote and support the adoption of new implementations, such as pharmacogenetic guided prescribing)* |
| 1 | Tension for Change | The degree to which stakeholders perceive the current situation as intolerable or needing change.  *(The demand from clinical and public stakeholder for pharmacogenetic guided prescribing – likely to be contextualised to different specialities)* |
| 2 | Compatibility | The degree of tangible fit between meaning and values attached to the intervention by involved individuals, how those align with individuals’ own norms, values, and perceived risks and needs, and how the intervention fits with existing workflows and systems.  *(Do stakeholders believe that pharmacogenetic guided prescribing will result in improved patient outcomes, without negatively impacting other areas of care?)* |
| 3 | Relative Priority | Individuals’ shared perception of the importance of the implementation within the organization.  *(Is pharmacogenetic guided prescribing seen as an important intervention at the organisation?)* |
| 4 | Organizational Incentives & Rewards | Extrinsic incentives such as goal-sharing awards, performance reviews, promotions, and raises in salary, and less tangible incentives such as increased stature or respect.  *(How is pharmacogenetic guided prescribing encouraged, rewarded, or incentivised?)* |
| 5 | Goals and Feedback | The degree to which goals are clearly communicated, acted upon, and fed back to staff, and alignment of that feedback with goals.  *(Is there a feedback loop built into the design of the pharmacogenetic programme, where stakeholder receive feedback on the development of the programme?)* |
| 6 | Learning Climate | A climate in which: a) leaders express their own fallibility and need for team members’ assistance and input; b) team members feel that they are essential, valued, and knowledgeable partners in the change process; c) individuals feel psychologically safe to try new methods; and d) there is sufficient time and space for reflective thinking and evaluation.  *(Is the institution used to implementing complex interventions and learning from these processes?)* |
| E | Readiness for Implementation | Tangible and immediate indicators of organizational commitment to its decision to implement an intervention.  *(Does the organisation have the resources and willingness in place to implement pharmacogenetics?)* |
| 1 | Leadership Engagement | Commitment, involvement, and accountability of leaders and managers with the implementation.  *(Has the design or inception of the pharmacogenetic programme been driven by the leadership team, with corresponding support?)* |
| 2 | Available Resources | The level of resources dedicated for implementation and on-going operations, including money, training, education, physical space, and time.  *(Have resources been made available (both in time and money) to support the design and maintenance of a pharmacogenetic programme?)* |
| 3 | Access to Knowledge & Information | Ease of access to digestible information and knowledge about the intervention and how to incorporate it into work tasks.  *(Do clinical stakeholders have easy access to the information required to support pharmacogenetic guided prescribing? These might include guidelines or educational resources)* |
| **IV. CHARACTERISTICS OF INDIVIDUALS** | |  |
| A | Knowledge & Beliefs about the Intervention | Individuals’ attitudes toward and value placed on the intervention as well as familiarity with facts, truths, and principles related to the intervention.  *(Do stakeholders believe that pharmacogenetic guided prescribing is useful to their practice and is well evidenced?)* |
| B | Self-efficacy | Individual belief in their own capabilities to execute courses of action to achieve implementation goals.  *(Do stakeholders believe that, given the available resources and support, that they can perform pharmacogenetic guided prescribing?)* |
| C | Individual Stage of Change | Characterization of the phase an individual is in, as he or she progresses toward skilled, enthusiastic, and sustained use of the intervention.  *(Are stakeholders familiar with using the pharmacogenetic programme at their institution?)* |
| D | Individual Identification with Organization | A broad construct related to how individuals perceive the organization, and their relationship and degree of commitment with that organization.  *(Do stakeholders want to support a new initiative, like pharmacogenetic guided prescribing, at their institution?)* |
| E | Other Personal Attributes | A broad construct to include other personal traits such as tolerance of ambiguity, intellectual ability, motivation, values, competence, capacity, and learning style.  *(Are clinical stakeholders motivated and interested to support the implementation of a pharmacogenetic guided prescribing programme?)* |
| **V. PROCESS** | |  |
| A | Planning | The degree to which a scheme or method of behavior and tasks for implementing an intervention are developed in advance, and the quality of those schemes or methods.  *(Has the pharmacogenetic programme been adequately planned and is there a framework for growing the initiative?)* |
| B | Engaging | Attracting and involving appropriate individuals in the implementation and use of the intervention through a combined strategy of social marketing, education, role modeling, training, and other similar activities.  *(Is there an appropriate team/network in place to develop the pharmacogenetic programme at the institution?)* |
| 1 | Intervention Participants | Individuals who will be impacted by the intervention.  *(Are public stakeholders involved in the design of the programme through public and patient engagement groups?)* |
| 2 | Opinion Leaders | Individuals in an organization who have formal or informal influence on the attitudes and beliefs of their colleagues with respect to implementing the intervention.  *(Are there key opinion leaders at an organisation who can drive implementation of pharmacogenetics?)* |
| 3 | Formally Appointed Internal Implementation Leaders | Individuals from within the organization who have been formally appointed with responsibility for implementing an intervention as coordinator, project manager, team leader, or other similar role.  *(Is there an administrative structure within the pharmacogenetic programme which can support and deliver pharmacogenetics?)* |
| 4 | Champions | “Individuals who dedicate themselves to supporting, marketing, and ‘driving through’ an [implementation]” [101] (p. 182), overcoming indifference or resistance that the intervention may provoke in an organization.  *(Have pharmacogenetic champions been appointed?)* |
| 5 | External Change Agents | Individuals who are affiliated with an outside entity who formally influence or facilitate intervention decisions in a desirable direction.  *(Are there key opinion leaders from outside the organisation who have had active engagement with the pharmacogenetic programme to support its implementation?)* |

**Table S1. CFIR Constructs and Definitions.** Extracted from the CFIR Research Team-Center for Clinical Management Research portal. Accessed via cfirguide.org on 01/02/2022.

| **Table S2. Pharmacogenetic Testing Programmes and Initiatives** | | | | | | | | | |
| --- | --- | --- | --- | --- | --- | --- | --- | --- | --- |
| **Country** | **Programme** | **Publications ^(References)^** | **Care Setting** | **Population** | **Patients Tested (n)** | **Genetic Testing** | **EHR Integration** | **EHR Provider** | **Eligibility for PGx Testing** |
| USA | Arkansas Children's Hospital, Little Rock, AR | 1^(1)^ | PC & SC | Paediatric | Not Described | 23 genes (173 variants) | Level 1-BPAs Implemented | EPIC | Prescription of a PGx medicine |
|  | Atrium Health Levine Cancer Institute, Charlotte, NC | 2^(2,3)^ | SC | Adult and Paediatric | 200 | 14 genes  (66 variants) | Level 4-Stand Alone Report | Not Described | Developmental disorder / oncology patients |
|  | Boston Children’s Hospital, Boston, MA | 1^(4)^ | SC | Paediatric | 396 | 225 genes  (1900 variants) | Level 1-BPAs Implemented | Cerner | Clinical Decision |
|  | Cincinnati Children's Hospital, Cincinnati, OH | 1^(5)^ | SC | Paediatric | 8,700 | 2 genes  (28 variants) | Level 2- Report Alerts | EPIC | Prescription of a PGx Medicine |
|  | Colorado Center for Personalized Medicine, Aurora, CO* | 1^(6)^ | PC & SC | Adult | 53,000 | 2.1 million variants | Level 1-BPAs Implemented | EPIC | Biobank study |
|  | Coriell Personalized Medicine Collaborative, Camden, NJ | 2^(7,8)^ | PC & SC | Adult | 4,372 | 1,936 variants | Not Described | Not Described | Observational cohort study |
|  | Duke University Health System, Durham, NC | 5^(2,9–12)^ | PC | Adult | Not Described | 225 genes (1900 variants) | Level 3-Report Uploaded to EHR | EPIC | Prescribed ≥3 medicines including simvastatin or clopidogrel |
|  | Elmwood Family Physicians, Evesham Township, NJ | 1^(13)^ | PC | Adult | 50 | 14 genes | Level 3-Report Uploaded to EHR | Not Described | Taking at least 7 medications |
|  | Eskenazi Health System, Indianapolis, IN | 3^(14–16)^ | SC | Adult | 6000 | 14 genes (43 variants) | Level 3-Report Uploaded to EHR | Not Described | Prescription of a PGx medicine |
|  | Innova Health System, VA | 1^(17)^ | SC | Paediatric | 4,257 | 7 genes | Level 3-Report Uploaded to EHR | Not Described | Newborn Screening [MediMap] |
|  | Mission Health, NC | 2^(18,19)^ | PC & SC | Adult | 51 | Not Described | Level 4- Stand Alone Report | Not Described | ≥age 65, taking ≥4 prescription medications, & class A or B interaction |
|  | NorthShore University Health System, Evanston, IL | 3^(20–22)^ | PC & SC | Adult | Not Described | Not Described | Level 3-Report Uploaded to EHR | Not Described | Clinical Decision |
|  | Ohio State University Medical Center, Columbus, OH | 2^(23,24)^ | PC & SC | Adult | Not Described | 1,936 variants | Level 3-Report Uploaded to EHR | Not Described | Not Described |
|  | PHARM-GENOME-PACE, NJ | 1^(25)^ | PC | Adult | 296 | 11 genes | Level 4-Stand Alone Report | N/A | >age 55 and enrolled in PACE |
|  | PHASER, VA Health Care System, Durham, NC | 2^(26,27)^ | PC & SC | Adult | Not Described | 12 genes (52 variants) | Level 3-Report Uploaded to EHR | Cerner | Clinical Decision |
|  | Sanford Health, Sioux Falls, SD | 2^(28,29)^ | PC | Adult and paediatric | 11,000 | 8 genes | Level 1-BPAs Implemented | EPIC | Clinical Decision |
|  | St. Jude Children’s Research Hospital, Memphis, TN | 3^(30–33)^ | SC | Paediatric | 1559 | 230 genes (1936 variants) | Level 1-BPAs Implemented | Cerner | Clinical Decision |
|  | The Mount Sinai Hospital, New York, NY | 3^(34–36)^ | SC | Adult | 1,500 | Not Described | Level 1- BPAs Implemented | EPIC | Bio*Me* participants prescribed PGx medicine |
|  | The RIGHT Study, Mayo Clinic, Rochester, MN | 11^(37–47)^ | PC & SC | Adult | >10,000 | >250 genes | Level 1- BPAs Implemented | EPIC | Clinical Decision and Mayo Clinical Biobank Participants |
|  | The University of Chicago, Chicago, IL (The ImPreSS Trial & The 1200 Patients Trial) | 6^(48–53)^ | SC | Adult | >3000 | 29 genes | Level 1- BPAs Implemented | EPIC | Varying eligibility criteria depending on specific trial |
|  | University of Colorado Executive Health Programme, Aurora, CO | 1^(54)^ | EH | Adult | Not Described | 27 genes | Level 3-Report Uploaded to EHR | Not Described | Clinical Decision |
|  | University of Florida Health, Gainesville, FL | 6^(55–60)^ | PC & SC | Adult and paediatric | 793 | 8 genes  (32 variants) | Level 1- BPAs Implemented | EPIC | Varying eligibility criteria depending on specific trial |
|  | University of Michigan, Ann Arbor, MI | 1^(61)^ | PC & SC | Adult | 200 | 15 genes | Level 1- BPAs Implemented | EPIC | Clinical Decision |
|  | University of Minnesota, Minneapolis, MN | 2^(62,63)^ | PC & SC | Adult and paediatric | Not Described | 20 genes (48 variants) | Level 3-Report Uploaded to EHR | EPIC | Clinical Decision |
|  | Outpatient Family Practice, University of Minnesota, Duluth, MN | 1^(64)^ | PC | Adult | 91 | Not Described | Level 3-Report Uploaded to EHR | Not Described | Prescription of a PGx medicine |
|  | University of Utah, Salt Lake City, UT | 1^(65)^ | PC & SC | Adult | 205 | 5 genes  (44 variants) | Level 4- Stand Alone Report | Not Described | Age >65 years, initiating new medicine and prescribed >2 medicines |
|  | Vanderbilt, Nashville, TN (PREDICT) | 8^(66–73)^ | PC & SC | Adult | >10,000 | 10 genes (45 variants) | Level 1- BPAs Implemented | EPIC | Clinical Decision |
| Canada | ICANPIC Study, Toronto, Ontario | 1^(74)^ | PC | Adult | 100 | 9 genes  (61 variants) | Level 4- Stand Alone Report | Not Described | Prescription of a PGx medicine |
|  | The Hospital for Sick Children, Toronto, Ontario | 1^(75)^ | SC | Paediatric | 172 | 6 genes | Level 3-Report Uploaded to EHR | Not Described | Prescription of a PGx medicine or Previous ADR |
|  | The IMPACT Trial, Toronto, Ontario | 1^(76)^ | PC & SC | Adult and paediatric | Not Described | 8 genes  (59 variants) | Level 4- Stand Alone Report | Cerner | Treated with psychotropic medication |
|  | University of British Columbia, Vancouver | 1^(77)^ | PC | Adults | 191 | 7 genes  (33 variants) | Level 4- Stand Alone Report + Independent Decision Support Tool | Not Described | Clinical Decision |
| Spain | La Paz University Hospital, Madrid, Spain | 1^(78)^ | SC | Adults | 600 | 38 genes (192 variants) | Level 3-Report Uploaded to EHR | Not Described | Clinical Decision – Triaged by PGx Service |
|  | MedeA Initiative, Badajoz, Spain  (*In Early Development*) | 1^(79)^ | PC & SC | Adults | Not Described | Not Described | Level 1- BPAs Implemented | Not Described | Not Described |
|  | PriME-PGx, La Princesa University Hospital, Madrid, Spain | 1^(80)^ | SC | Adults | Not Described | 31 genes | Level 3-Report Uploaded to EHR | Not Described | Clinical Decision |
| Netherlands | Alliance Healthcare Community Pharmacy | 1^(81)^ | PC | Adults | 611 | 27 genes (111 variants) | Level 3-Report Uploaded to EHR | Not Described | Clinical Decision or Patient Request |
|  | The Implementation of Pharmacogenomics into Primary care Project (IP3), Leiden | 2^(82,83)^ | PC | Adults | 200 | 8 genes | Level 1- BPAs Implemented | G-Standard | Prescription of a PGx medicine |
| Thailand | Bumrungrad International Hospital, Bangkok | 1^(84)^ | SC | Adults | Not Described | Not Described | Level 1- BPAs Implemented | Not Described | Clinical Decision |
| Switzerland | Zurich Clinical Pharmacology | 1^(85)^ | SC | Adults | 135 | 16 genes | Level 4- Stand Alone Report | Not Described | Clinical Decision |
| New Zealand | Auckland Mental Health Services, Auckland | 1^(86)^ | SC | Adults | 23 | 2 genes | Level 4- Stand Alone Report | Not Described | Incident Prescription of Risperidone |
| China | Xiangya Hospital of Central South University, Changsha | 1^(87)^ | SC | Adults | 12,758 | 4 genes | Level 1- BPAs Implemented | Not Described | Clinical Decision |

**Table S2. Pharmacogenetic Testing Programmes and Initiatives.** PC **=** Primary Care**.** SC = Secondary Care. BPA**s =** Best Practice Advisories. Pediatrics care is defined as under 16 years of age. The remaining manuscripts described the multi centre IGNITE, e-MERGE, PGRN and U-PGX initiatives.

**Figure S1.**

**A)**


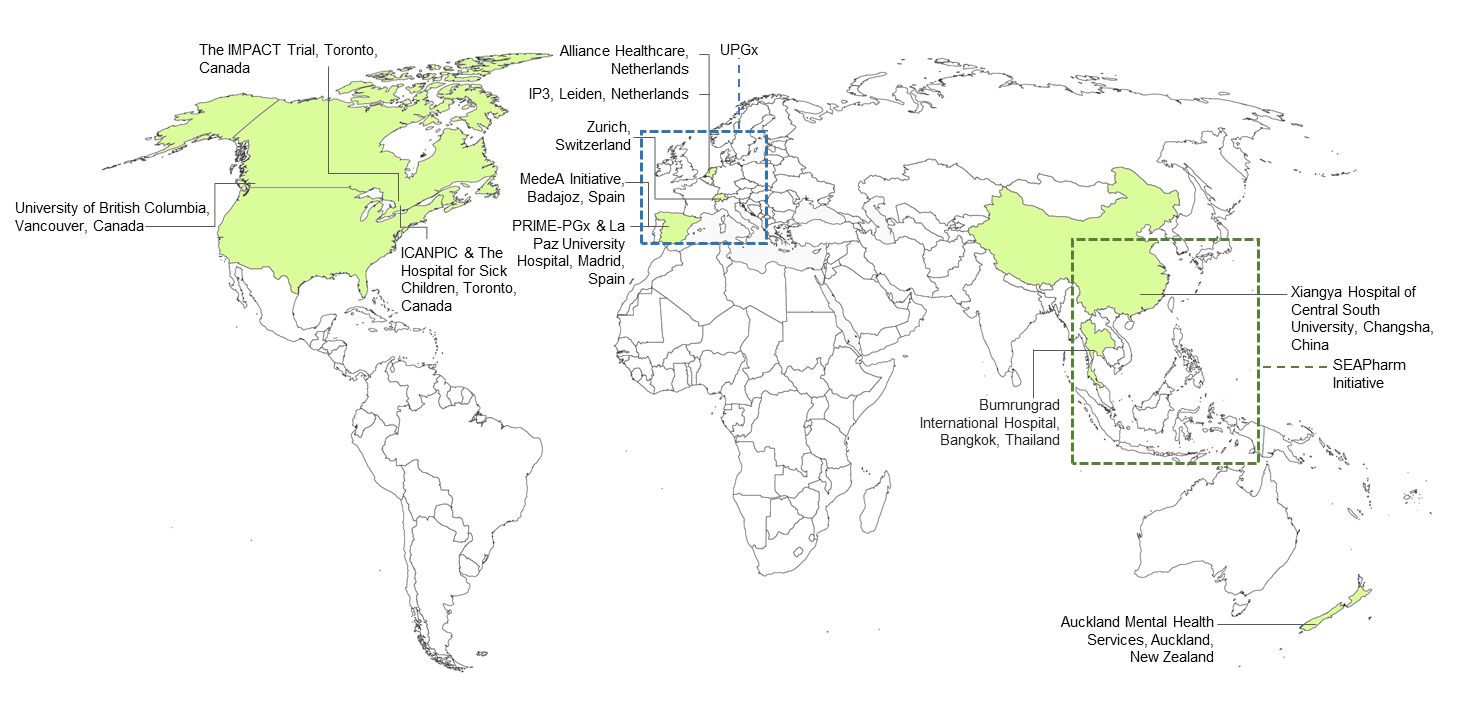


**B)**


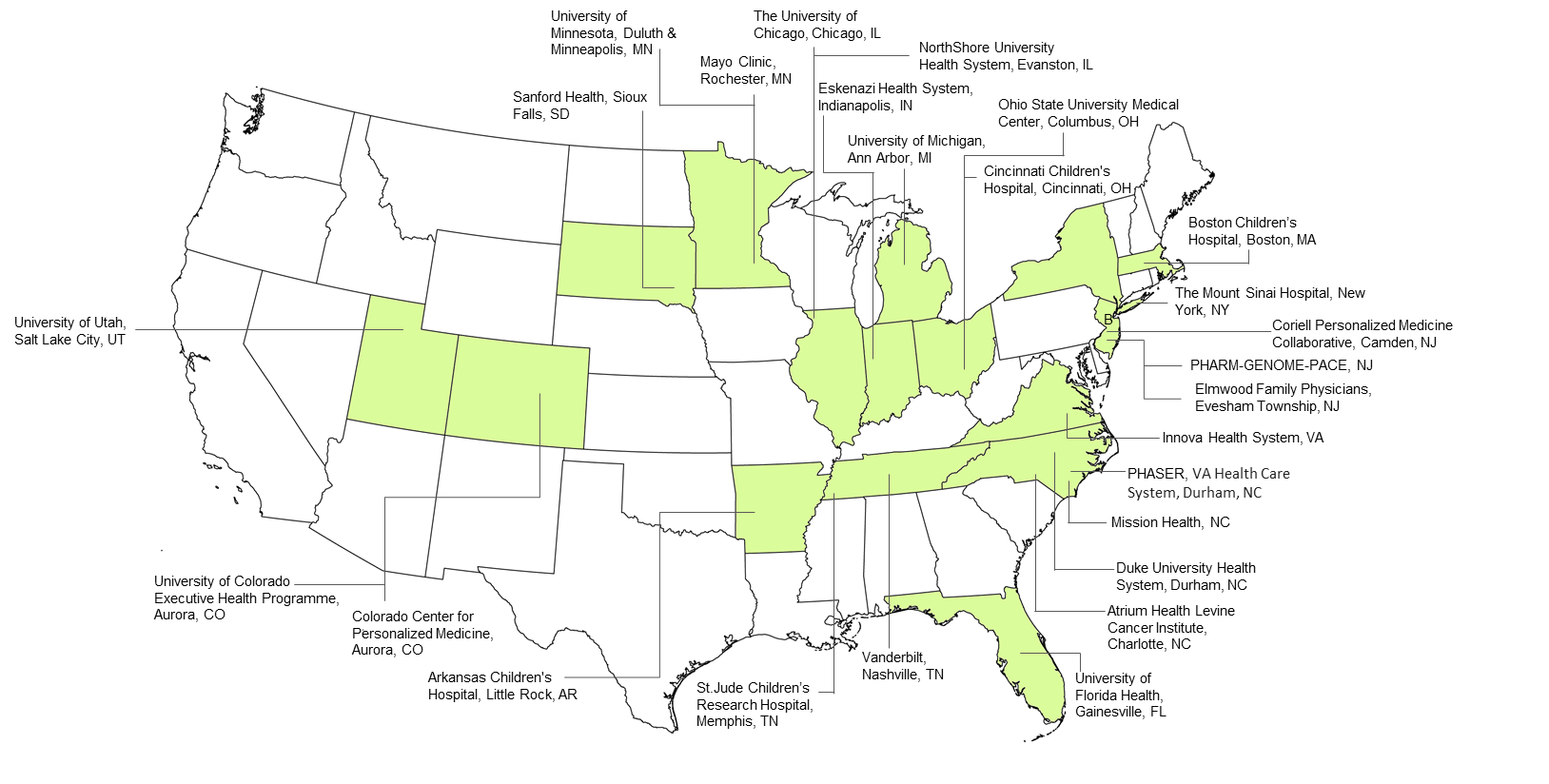


**Figure S1. Geographical Distribution of Pharmacogenetic Programmes.** (A) Global distribution of Pharmacogenetic Programmes and (B) distribution of programmes in the United States.

**Figure S2**


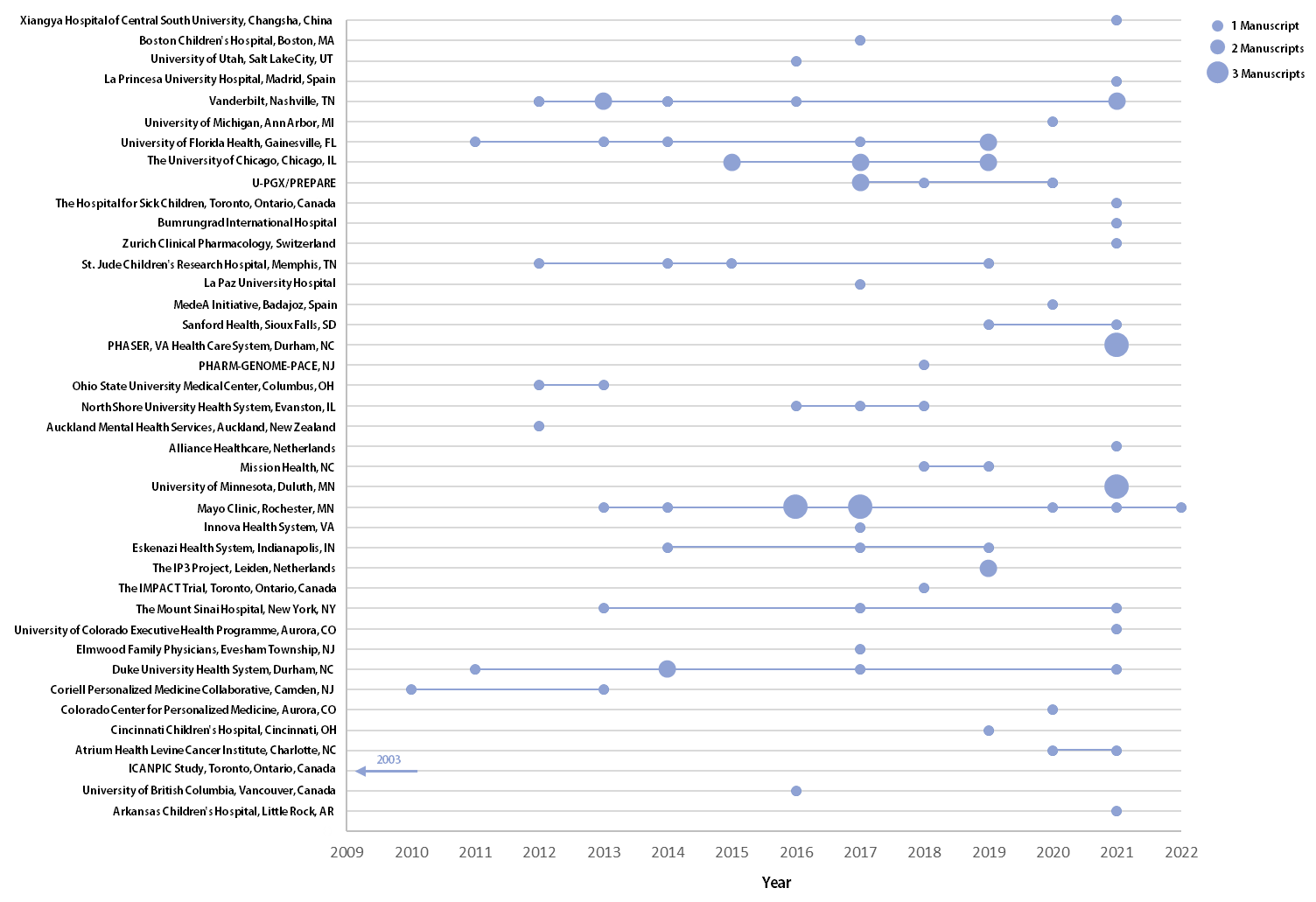


**Figure S2 Chronology of the Pharmacogenetic Literature.** Manuscripts published at each pharmacogenetic programme over time.

**Figure S3**

**
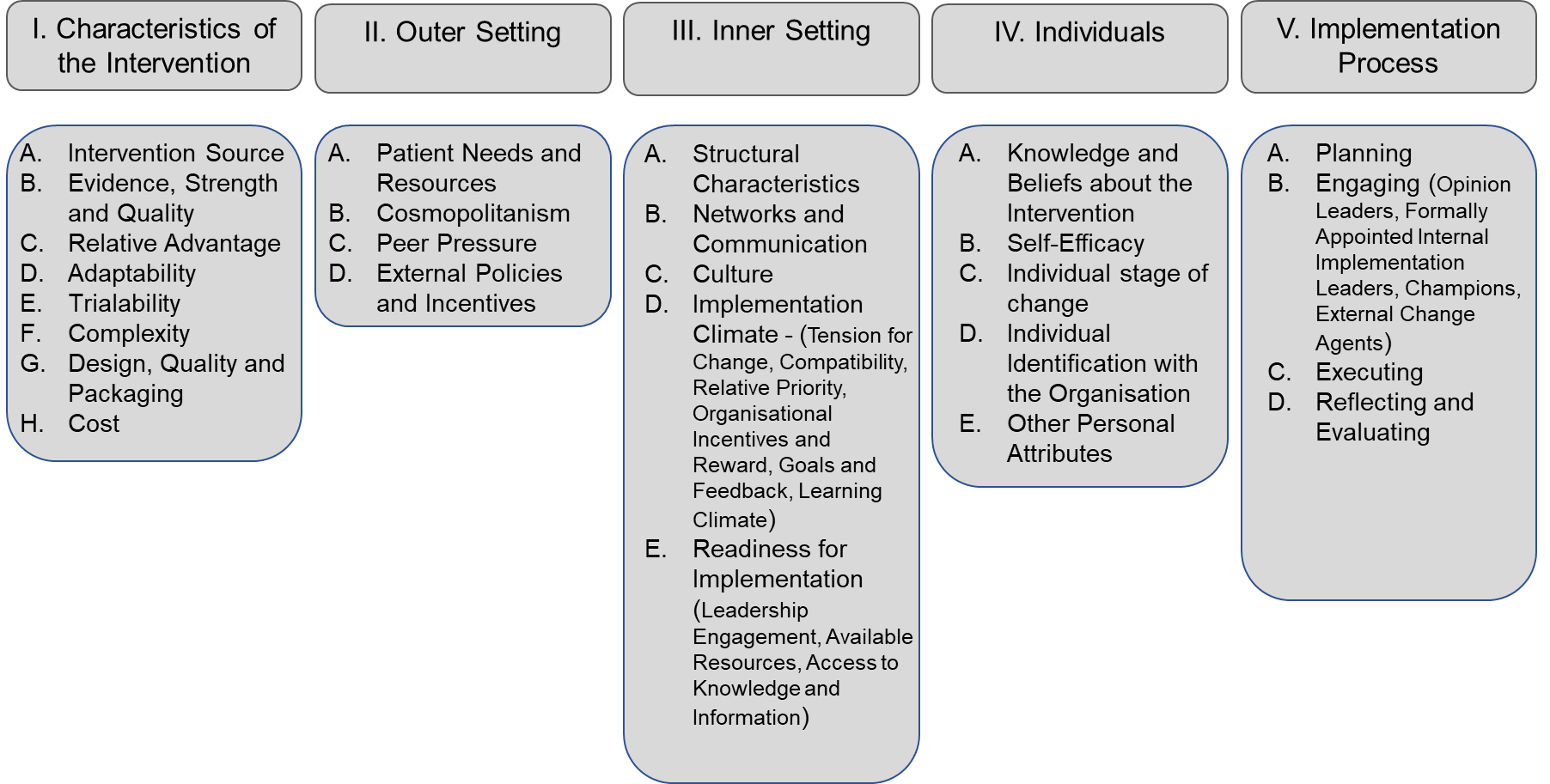
**

**Figure S3. The Consolidated Framework for Implementation Research.** Associated Domains and Constructs.
